# Traditional and sustainable approaches for the construction of C–C bonds by harnessing C–H arylation

**DOI:** 10.1038/s41467-022-28707-9

**Published:** 2022-02-28

**Authors:** Jagrit Grover, Gaurav Prakash, Nupur Goswami, Debabrata Maiti

**Affiliations:** Department of Chemistry, Indian Institute of Technology Bombay, Powai, Mumbai, 400076 India

**Keywords:** Homogeneous catalysis, Synthetic chemistry methodology

## Abstract

Biaryl scaffolds are found in natural products and drug molecules and exhibit a wide range of biological activities. In past decade, the transition metal-catalyzed C–H arylation reaction came out as an effective tool for the construction of biaryl motifs. However, traditional transition metal-catalyzed C–H arylation reactions have limitations like harsh reaction conditions, narrow substrate scope, use of additives etc. and therefore encouraged synthetic chemists to look for alternate greener approaches. This review aims to draw a general overview on C–H bond arylation reactions for the formation of C–C bonds with the aid of different methodologies, majorly highlighting on greener and sustainable approaches.

## Abstract (web summary)

Transition-metal-catalyzed C–H arylations are an effective tool for the construction of biaryl motifs in an efficient and selective manner. Here the authors provide an overview of the state-of-the-art of the field and perspectives on emerging directions toward increased sustainability.

## Introduction

Over the last two decades, cross-coupling reactions have had a scintillating effect on medicinal chemistry, drug discovery, and agrochemicals, etc. These methods are easy to perform, reliable, and quickly generate an assay of chemical space of different structural scaffolds. One of such widespread cross-coupling reactions is Suzuki-Miyaura cross-coupling (Nobel Prize in 2010), which has been extensively studied by researchers in academia as well as in industry for synthesis of biaryls and hetero-biaryls^1^. A study reveals that Suzuki coupling is the 2nd most utilized chemical transformation in medicinal chemistry to synthesize several drug molecules^2^. Suzuki coupling strategy demands a pre-functionalized (hetero)arenes as substrate and arylboronic acid as coupling partner. Over the years, several modified versions of Suzuki coupling have been discovered^3^. The use of pre-functionalized arenes sought to be one of the significant drawbacks of this methodology^4^. In this context, direct C–H functionalization emerged as an efficient tool in the construction of C–C bonds. C–H arylation omits the use of pre-functionalized arenes, thus reflecting its superiority over cross-coupling strategies. However, controlling the site selectivity during C–H activation has always been a complicated issue to address. The problem of site selectivity has been addressed up to some extent with the help of directing group-assisted transition-metal-catalyzed C–H functionalization. Both aliphatic and aromatic C–H bonds were arylated with the help of a suitable directing group attached with the molecule^5^. Later on, various other methods for site-selective C–H functionalization were developed. Transition-metal-catalyzed C–H arylation has been well explored over decades but suffers from major drawbacks *viz* harsh reaction conditions (elevated temperature, additives) and the use of expensive reagents, in turn diminishing the utility of such methodologies. Therefore, a sustainable approach was required to address such challenges. In this context, photoredox C–H arylation^6,7^ and electrochemical C–H arylation^8,9^ came up as other available options. Pd/photocatalyst merger can achieve the regioselective arylation of arenes and heteroarenes at room temperature. Electrochemical C–H functionalization makes use of earth-abundant 3d metals and employs electricity as the oxidant, which makes the overall transformation economic and eco-friendly. Traditional transition-metal-catalyzed processes usually have longer reaction time which limits the industrial utility of such functionalizatios^10,11^ turned out as an economical pathway to carry out such type of transformations in a cleaner and faster fashion.

Reduced reaction time, cleaner product formation, safer reaction protocol, and easy scale-up are the key advantages of flow process. Furthermore, with increasing interest in renewable source of energy, chemists are striving to eliminate the waste and recycle them to achieve the results through greener pathways. Therefore, the area of mechanochemistry^12,13^ which includes grinding along with sonication, ball milling, etc., is of great interest to the researchers working toward developing greener methodologies.

Mechanochemical reaction proceeds in the absence of excess solvents/no solvent or heating, making this a vital component of the recent surge in interest toward green chemistry. Apart from these methodologies, usage of 3d base metals for C–H functionalization also contributes toward sustainable approaches. Since, they feature in many biological processes and are also less toxic than their corresponding 4d and 5d, analogs^14^. Their abundance in Earth’s crust is also high, makes them cost-efficient catalysts. All these strategies altogether have broadened up the spectrum of C–H arylation in terms of sustainability and applicability in the area of organic synthesis.

### Directing group-assisted transition-metal-catalyzed C(*sp*^2^)–H arylation

**Fig. 1:**
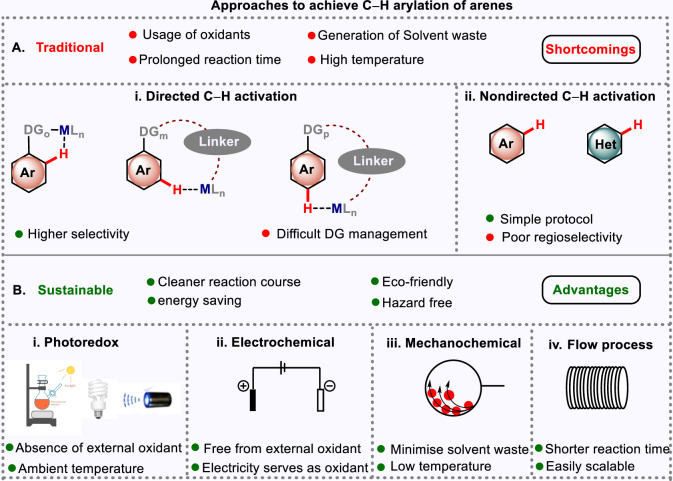
Approaches for site-selective C–H arylation. **A** Traditional approaches of C–H arylation of arenes. **B** Sustainable approaches of C–H arylation of arenes.

Selective functionalization of identical and chemically equivalent C–H bonds has always been in the prime focus of synthetic chemists. In this regard, the use of transition-metal catalysts normalizes the pre-existing barrier to prevail a selective C–C bond formation Fig. 1. A suitably designed directing group can coordinate with the transition metal to form a cyclometallated intermediate, thus reaching in the vicinity of the target C–H bond to avail a successful functionalization^15^. Directing groups known so for primarily can be classified into two types, i.e., strong co-ordinating like pyridine^16^, pyrimidine^17^ and weakly co-ordinating like acid^18^, amide^19^, nitrile^20^. The initial study was commenced with strong directing groups. However, later weak directing groups also captured significant attention, as a number of C–H functionalizations were achieved with help of these directing groups. Functionalization at proximal positions, such as *ortho-*functionalization in aromatic systems and *β-*functionalization in aliphatic systems are relatively easier as compared to distal positions (*meta-, para-, γ,* and *δ-*) due to the formation of thermodynamically favorable five-membered metallacycle while later proceeds through formation of a strained metallacycle.

### *Ortho*-C–H arylation

**Fig. 2:**
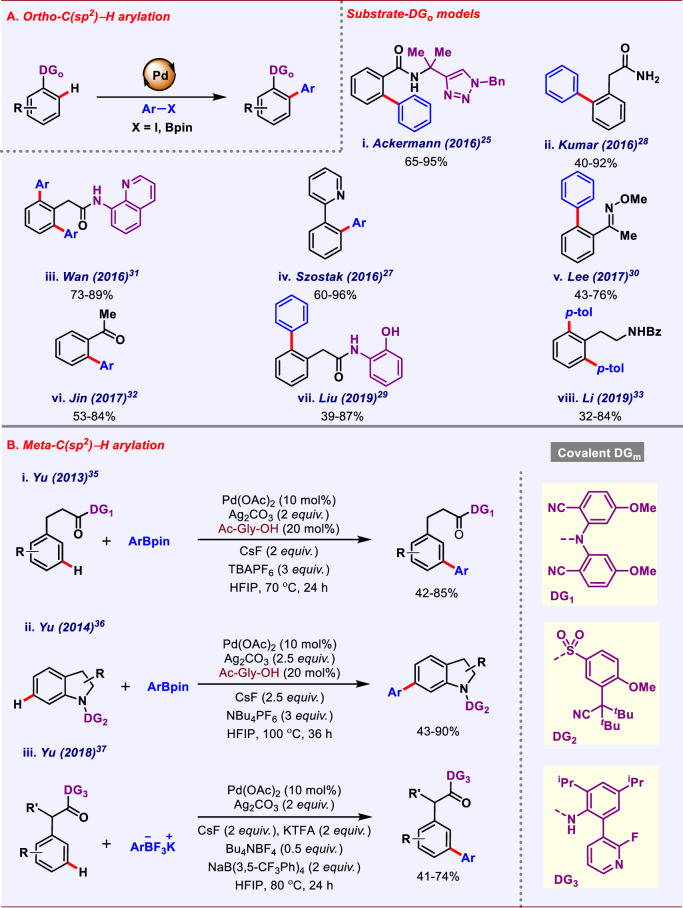
Covalent directing group-assisted C(*sp*^2^)–H arylation of arenes. **A** *ortho*-C(*sp*^2^)–H arylation of arenes. **B** *meta*-C(*sp*^2^)–H arylation of arenes.

Different strategies for *ortho*-C–H arylation have been devised. However, the most applicable among them is directing group-assisted *ortho*-C–H arylation via the formation of five to six-membered metallacycle, which is thermodynamically favorable. Many 4d transition metals (Pd, Rh, Ru,) along with 3d transition metals (Cu, Co, Ni, Fe) are very well-known for the realization of *ortho*-C–H functionalization. However, palladium catalysis is leading and holding the regime of *ortho*-C–H activation. In this field pioneer works were performed by Yu^21^, Dauglis^22^, and Sanford^23^. The Dauglis group in 2005 reported *ortho-C*–H arylation of anilides using diaryliodonium salts as the coupling partner^22^. However, due to lesser availability and expensive nature of iodonium salts, only limited scope could be performed. Later, Yu group in 2008 also performed *ortho*-C–H arylation of benzoic acids and phenylacetic acids utilizing aryl trifluoroborate as the coupling partner^21^. Various kinds of substituents, i.e., electron-donating as well as electron-withdrawing were well-tolerated. Numerous works have been reported on *ortho*-C–H arylation catalyzed by transition metals and is already covered in previously reported reviews^24^. Herein we have mainly focused on only recent advances in *ortho*-C–H arylation realized by using palladium catalyst. In 2016, Ackermann and co-workers reported Pd catalyzed *ortho*-C–H arylation of benzamides using triazole as the directing group (Fig. 2A, i)^25^. The reaction proceeded with Pd(OAc)_2_ as the catalyst, AgOAc as oxidant, and aryl iodide as the coupling partner. Later 2017, Watkins and co-workers extended this protocol for *ortho-*C–H arylation of benzamides^26^. Amino acetanilide being a bidentate directing group, facilitates this *ortho-*C–H arylation in the presence of Pd catalyst. In 2016, Szostak and co-workers came up with *ortho*-C–H arylation of 2-phenylpyridines, where they utilized aryl amide as the arylating agent. (Fig. 2A, iv)^27^. Use of cheap and bench stable aryl amide further increased the worth of this protocol. In the same year, Kumar and co-workers came up with the first report of *ortho*-C–H arylation of arylacetamide^28^, where acetamide itself acted as a directing group (Fig. 2A, ii). Similarly, Liu and co-workers in 2019 executed tuneable mono- and di-*ortho-*C–H arylation of phenylacetamides^29^ enabled by *o*-aminophenol as the directing auxiliary. The absence of the hydroxyl group was detrimental for this *ortho-*C–H arylation (Fig. 2A, vii). In 2017, Lee and co-workers performed *ortho-*C–H arylation of acetophenone oxime ethers using aryl pinacol boronic ester as the arylating agent (Fig. 2A, v)^30^. Bench stability of aryl pinacolone boronate (ArBpin) and ease to handle, raised the worth of this protocol over previously known methods. In 2016, Wan and co-workers described domino *ortho*-C–H arylation of phenylacetamides in one pot (Fig. 2A, iii)^31^. In situ installation of 8-aminoquinoline as the directing group improved the step economy of the overall transformation. Mono- *vs* di-selectivity was controlled using base additive; however, they failed to explain the reason behind the achieved selectivity. Contemporarily, Jin and co-workers demonstrated *ortho*-C–H arylation of aromatic ketones^32^ enabled by glycine as a transient directing group (Fig. 2A, vi). Mechanistic studies concluded that C–H activation might be the rate-determining step for this transformation. Following this, Li and co-workers reported ligand-enabled *ortho*-C–H arylation of free phenethylamine (Fig. 2A, viii)^33^. Native -NH_2_ group present in the substrate itself acted as the directing group, thus increasing atom and step economy of the overall transformation.

### *Meta*-C(*sp*^2^)–H arylation

Reaching out to distal positions of arene systems has always been imperative to the research field. However, overriding the inherent electronic properties of the substrate is more challenging than proximal C–H functionalizations. This is mainly due to the ring strain and thermodynamic properties associated with the formation of 12–13-membered metallacycle intermediate. Interestingly, Yu group designed a U-shaped template that led to various types of *meta*-C**–**H functionalizations^34^. In 2013, Yu and co-workers reported the first example of *meta-*C–H arylation of phenylpropanoic acid and phenol derivatives enabled by a U-shaped covalently bonded template (Fig. 2B, i)^35^. With the application of perfect distance-geometry correlation, they judiciously designed the *meta*-directing unit utilizing the weak metal-ligand coordination of cyanide-containing tethering group, which can lead to the targeted distal-C–H bond activation. The use of monoprotected amino acid (MPAA) in catalytic amounts was found to act as both ligand as well as an internal base in the course of the reaction. Other reagents like surfactant and additive also played a crucial role in achieving *meta-*selectivity preferentially over other C–H bonds. In 2014, the same group obtained *meta-*C–H arylation of indolines^36^ driven by the same strategy (Fig. 2B, ii). To overcome the electronic bias in indoline derivatives, a novel template was designed that reduced the electron density inside the ring by its electron pulling nature. In 2018, Yu group reported *meta-*C–H arylation of phenylacetic acids using a pyridine-based template (Fig. 2B, iii)^37^. The previously designed cyanide template was unable to promote this transformation. However, pyridine-based template was found to be successful to perform this reaction.

### Norbornene as transient mediator

**Fig. 3:**
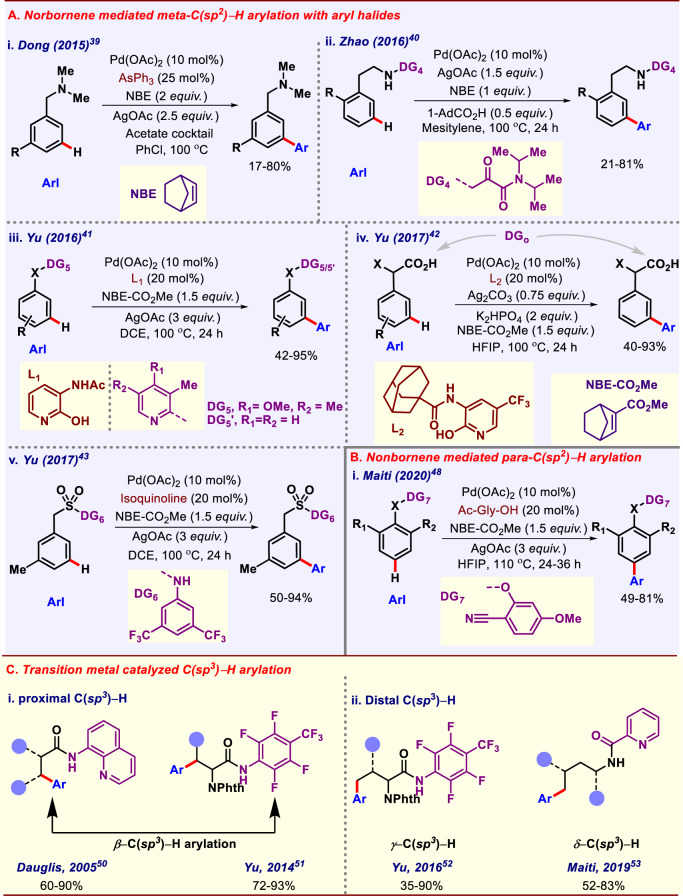
Transition-metal-catalyzed aromatic and aliphatic C–H arylation. **A** Norbornene mediated *meta*-C(*sp*^2^)–H arylation. **B** Norbornene mediated *para*-C(*sp*^2^)–H arylation. **C** Transition metal-catalyzed C(*sp*^3^)–H arylation.

Norbornene as a transient mediator relays the initial metallacycle to adjacent position via (Catellani reaction). Palladium forms 5–6-membered metallacyle, which reacts with norbornene to provide an intermediate, followed by the activation of *meta*-C–H bond and subsequently the aryl coupling partner forms new C–C bond then *β*-carbon elimination of norbornene and protodemetallation of the aryl–palladium bond regenerates the Pd catalyst^38^. In 2015, Dong group reported *meta-*C–H arylation of *N,N-*dimethylbenzylamine (Fig. 3A, i) using norbornene as a transient mediator^39^. Amine directing group can be easily installed and removed and can also be transformed into various other functional groups. In 2016, Zhao group reported first bidentate group assisted *meta-*C–H arylation of *β-*arylethylamine using norbornene as a transient mediator (Fig. 3A, ii)^40^. The directing group assists the metal to activate *meta*-C–H bond with the help of NBE mediator. Electron-donating as well as electron-withdrawing groups were well-tolerated under this protocol. In 2016, Yu group also reported *meta-*C–H arylation of anilines, phenols, and heterocycles following the same strategy (Fig. 3A, iii)^41^. Ligands played a very significant role in achieving *meta*-selectivity. In 2017, the same group again performed *meta-*C–H arylation of phenylacetic acids using the same relay strategy (Fig. 3A, iv)^42^. The native functional group -COOH itself acted as the directing group, which directs the metal to *ortho-*C–H of arene, NBE-CO_2_Me acting as a transient mediator, relayed this *ortho*-cyclopalladation to *meta-*C–H of the arene. In the same year, Yu group also reported ligand-enabled *meta*-C–H arylation of benzene sulfonamides^43^ (Fig. 3A, v). Benzyl sulfonamides are versatile functional groups that can be easily transformed into sulfonamide, sulfone, and alkene. Thus, C–H arylation of benzyl sulfonamides possesses a broad utility in drug discovery and styrene synthesis. Several reports on *para-*C–H arylation have been achieved through electronic biasness in the substrate that lead to moderate selectivity in the final product^44–47^. In this sense, the directing group-assisted approach would be more desirable. Hypothetically, template-mediated *para-*C–H functionalizations are difficult to accomplish due to formation of thermodynamically unstable 17–18-membered metallacycle intermediate. Maiti and co-workers first developed a D-shaped biphenyl nitrile template which can bind with metal and was able to selectively activate the remote *para-*C–H bond in the arene ring^20^. Very recently, Maiti and co-workers reported *para*-selective arylation of arenes using norbornene as a transient mediator (Fig. 3B, i). The goal to reach the remote *para-*C–H bond was accomplished by using 2-cyano-5-methoxyphenol as a *meta*-directing group and 2-carbmethoxy norbornene as a transient mediator^48^. It was observed that only electron-deficient aryl iodides were suitable for this transformation. A gram scale reaction along with sequential C–H olefination manifested the importance of this transformation.

### Directing group-assisted C(*sp*^3^)–H arylation

Activation of C(*sp*^3^)–H bond is a synthetically challenging task as compared to the activation of C(*sp*^2^)–H bond, owing to its inertness. Many groups around the globe have tried to functionalize C(*sp*^3^)–H bond regio-selectively with the aid of covalently bonded directing groups^49^. Different types of mono- and bidentate groups are used as the directing auxiliary. Among all of them, 8-aminoquinoline is the widely exploited directing template for aliphatic C–H functionalization. The first report of C(*sp*^*3*^)–H arylation of amides using 8-aminoquinline as directing group hails from Daugulis group (Fig. 3C, i)^50^, where they used aryl Iodide as coupling partner, Pd(OAc)_2_ as catalyst and AgOAc as an oxidant. This protocol was compatible with various functional groups present in the substrates. Apart from 8-aminoquinoline, other directing templates such as picolinamide, pyridine, oxazole, triazole, pyrazole, 2-aminopyridine-*N*-oxide and some *N,S*-derived bidentate directing groups were also utilized for proximal C(*sp*^*3*^)–H arylation for different aliphatic substrates. In 2014, the Yu group devised a method for ligand-controlled *β*-C(sp^3^)–H arylation in alanine derivative with aryl iodides^51^. This protocol paved a route for synthesis of unnatural chiral α-amino acids.

Groups such as the Daugulis, the Carratero, and the Yu made use of different auxiliaries for *γ*-C(*sp*)^3^–H arylation (Fig. 3C, ii)^52^. Not only 4d metals like Pd, Rh but 3d metals such as Ni, Fe were also used for aliphatic C–H arylation^14^. However, all these arylation reports were limited to up to gamma position of the aliphatic substrates.

In 2019, Maiti and co-workers demonstrated the (hetero)arylation of amino acids and analogous aliphatic amines selectively at the distal *δ*-position by varying the ligand to control the reactivity (Fig. 3C, ii)^53^. Later on, C(*sp*^3^)–H arylation using transient directing group such as imine^54^ and native functional group (COOH, NH_2_, etc.) as the directing group were developed by Yu^55,56^, Maiti^57^, and Bannister^58^ groups. However, discussion of those works is beyond the scope of this review.

### Transition-metal-catalyzed asymmetric C–H arylation

**Fig. 4:**
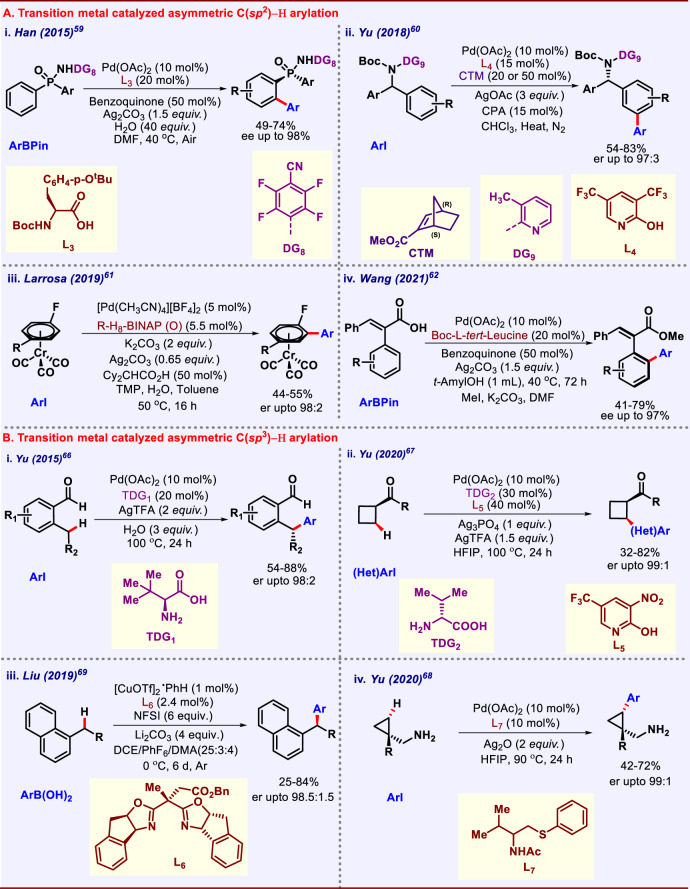
Transition-metal-catalyzed asymmetric C–H arylation. **A** Asymmetric C(*sp*^2^)–H arylation. **B** Asymmetric C(*sp*^3^)–H arylation.

Asymmetric C–H bond functionalization reactions can pave new paths for construction of chiral ligands which are widely used in asymmetric catalysis. Enantioselective C(*sp*^2^)–H arylation is mainly used to construct axial and planar chiral compounds. In this regard, Han and co-workers in 2015 executed desymmetric *ortho*-C–H arylation of diarylphosphinamides using arylboronic-ester as the arylating agent (Fig. 4A, i)^59^. Phosphorous-containing compounds have prime importance in transition-metal catalysis as well as organo-catalysis. Therefore, synthetic derivatization of these compounds is really an important achievement. Chiral amino acid ligand played the critical role in achieving this enantioselectivity. Particularly, acid group of amino acid was crucial. Various diarylphosphinamides were employed under this protocol releasing the desired *ortho-*arylated product with good yields and excellent enantiomeric excess. Yu group in 2018 devised a special approach for asymmetric functionalization of remote *meta*-C–H bond (Fig. 4A, ii)^60^. Asymmetric activation at remote positions is a difficult job to perform. Herein, they used combination of chiral transient mediator, i.e., nonbornene derivative and chiral phosphoric acid additive. These combined results revealed that the nonbornene decides the chirality of compound supported by chiral phosphoric acid ligand. This asymmetric transformation was realized by non-asymmetric C–H activation followed by stereo-selective nonbornene insertion. In 2019, Larrosa and co-workers developed a protocol for asymmetric arylation of (*η*^6^-arene)-chromiumtricarbonyl complexes using chiral BINAP type of ligands. A variety of iodoarenes and chromiumtricarbonyl complexes were well-tolerated giving the desired products in good yield and excellent enantioselectivity (Fig. 4A, iii)^61^. Mechanistic studies suggested that the reaction proceeds through Pd–Ag bimetallic complex, in which Ag is responsible for C–H activation. Very recently Wang and co-workers demonstrated synthesis of styrene-type chiral carboxylic acids (Fig. 4A, iv)^62^. These types of chiral carboxylic acids are majorly used as chiral ligands in transition-metal catalysis or as organocatalysts, this clearly reveals the importance of this protocol. A broad substrate scope with good yields and excellent enantio-control was achieved under mild conditions that reflected the robustness of this protocol. Formed chiral carboxylic acids were also applied to cobalt catalyzed asymmetric C–H activation as potential chiral ligands. Same kind of atroposelective arylations using other transition metals such as Rh and Ir are accomplished by groups of You, Crammer, and Wozniak^63–65^. Asymmetric C(*sp*^*3*^)–H arylation is mainly realized by transient directing group approach. Transient directing group (TDG) allow to perform the C–H functionalization by circumventing the two steps, i.e., installation of DG prefunctionalization and removal of DG post-functionalization. However, this strategy has its own limitations. In transient directing group mediated C–H functionalization, the most commonly used TDG are *N*-protected amino acids. Therefore, in such cases another external base is needed. Apart from that, the site of C–H bond scission is far from the stereogenic center in the TDG strategy. In 2015, Yu and co-workers performed asymmetric C–H arylation at methylene C(*sp*^*3*^)–H bond of benzaldehyde derivatives using a chiral amino acid as transient directing group. The reaction proceeds via the formation of imine in between benzaldehyde and L-*tert* leucine (Fig. 4B, i)^66^. Under optimized conditions, different aryl coupling partners were varied to achieve high enantiomeric ratio. Yu group also performed enantioselective arylation of free carboxylic acid in 2018 using cyclopropane carboxylic acid as model substrate and chiral monoprotected aminoethyl amine ligand^55^. Enantioselective *β*-C(*sp*^*3*^)–H arylation of aliphatic ketones using D-valine as chiral transient directing group were developed by the same group in 2020. A range of ketones containing cyclobutane derivatives were arylated using wide array of (hetero)aryl iodides. 3-Nitro-5-trifluoromethyl-2-pyridone promoted the C–H bond cleavage while its combination with silver salt was crucial in achieving high enantioselectivity (Fig. 4B, ii)^67^. They also performed a bidentate chiral thioether-based ligand-controlled *γ*-C(*sp*^*3*^)–H arylation of free cyclopropylmethylamine. Thioether motif favour the generation of amine-Pd(II) intermediate, which in turn enables the enantioselective C–H arylation of free amines (Fig. 4B, iv)^68^. Asymmetric benzylic C–H arylation of alkyl arenes by copper-catalyzed radical relay was realised by Liu and co-workers. Benzyl ester moiety containing bisoxazoline (Box) ligand was found to be the most effective in terms of enantioselectivity and yield. Authors also performed post synthetic modifications of Splitomicin and further converted to ring-opened amination products (Fig. 4B, iii)^69^. Wencel-Delord, Colobert, and co-workers developed an unprecedented chiral *N*-protected aminosulfoxides for direct enantioselective functionalization of unbiased C(*sp*^3^)–H bonds. An array of electron-deficient and electron-rich aryl iodides was well versed under this protocol. DFT calculations support the unique activity of the auxiliary, which led to almost barrier-less palladation event^70^. So far, many other groups have contributed toward enantioselective C–H arylation^71^ and discussion of all those works is beyond the scope of this review.

### Photoredox C–H arylation of arenes and heteroarenes

Direct C–H arylation of arenes and heteroarenes through photoredox catalysis emerged as an attractive alternative for traditional transition-metal-catalyzed C–H arylation. It removes the necessity to use external oxidants and proceeds under milder conditions. So, the overall process becomes greener as a lesser amount of metal catalyst is required. Generally, the aryldiazonium salt is used as an aryl coupling partner due to its lower reduction potential^72^. Later on, aryl iodonium salts, sulfonium salts, and aryl halides were also employed as aryl coupling partners in photoredox catalysis. Ru- and Ir-based inorganic salts are commonly used as photocatalysts. 2,2′-Bipyridine (bpy)-containing ruthenium complexes such as [Ru(bpy)_3_]^2+^ are considered to be suitable photocatalysts since they have absorption maxima at 452 nm (blue light), along with high chemical stability and excellent quantum yield.

### Metallaphotoredox C(sp^2^)–H arylation

**Fig. 5:**
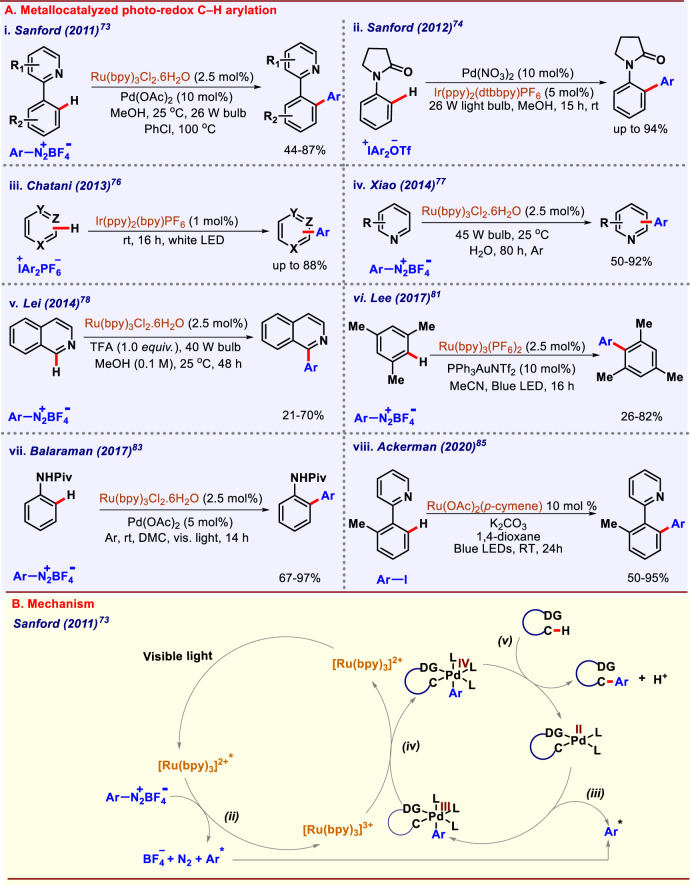
Photoredox catalyzed C–H arylation. **A** Metallacatalyzed photoredox C–H arylation. **B** Mechanism of ruthenium photocatalyzed arylation of phenylpyridines.

In 2011, Sanford group was the first to report Pd/Ru dual catalyzed C–H arylation under visible-light photoredox conditions (Fig. 5A, i)^73^. C–H arylation of arenes were performed using aryldiazonium salts as coupling partner in the presence of Pd(OAc)_2_, Ru(bpy)_3_Cl_2_.6H_2_O, and 26 W visible-light source. They used a variety of *ortho*-directing groups such as amides, pyrazoles, oxime ethers, and pyrimidines for *ortho*-C–H arylation. The reaction was proposed to follow the catalytic cycle shown in (Fig. 5B). First, Ru catalyst gets excited in the presence of visible light to generate Ru(bpy)^32+*^; then aryl radical was generated by reduction of aryldiazonium salt and concomitant oxidation of the Ru center takes place to generate Ru(bpy)^3+^. Subsequently, aryl radical reacts with palladacycle and produces Pd^III^ intermediate. One-electron oxidation by Ru(bpy)_3_^3+^ regenerates the photocatalyst and forms Pd^IV^ intermediate. The C–C bond formation occurs through reductive elimination to release the arylated product and regenerates the Pd^II^ catalyst. Apart from aryldiazonium salts, other aryl coupling partners such as iodonium, and sulfonium salts are effective under photoredox conditions. Later, the same group reported Pd/Ir-catalyzed C–H arylation with diaryliodonium reagents using a 26 W light bulb for 15 h at room temperature (Fig. 5A, ii)^74^. Mechanistic studies revealed that Ph_2_I^+^ generates aryl radical under visible-light irradiation in the presence of the photocatalyst. Diverse arene substrates were well-tolerated with a variety of iodonium salts. The reaction was supposed to follow a merged Pd^II/IV^ and Ir^III/IV^ catalytic cycles. Xiao’s group demonstrated room temperature arylation of arenes and heteroarenes with diaryliodonium salts. They performed direct arylation of *N-*methylpyrrole, furan, thiophene, 2,3-benzothiophene, and 2,3-benzofuran using diaryliodonium salts^75^. Chatani and co-workers developed photocatalyzed arylation of (hetero)arenes with diaryliodonium salts in the presence and absence of photocatalyst (Fig. 5A, iii)^76^. [Ir(ppy)_2_(bpy)]PF_6_ photocatalyst was essential when benzene and other heteroarenes were subjected for arylation. On the contrary, photocatalyst was no longer required when pyrrole derivatives were used as substrate. However, photo-induced single electron transfer to Ar_2_I^+^ was responsible for initiating both these processes to generate aryl radical. Xiao and co-workers executed photocatalyzed arylation of electron-deficient heteroarenes with aryldiazonium salts in 2014 (Fig. 5A, iv). They reported radical-based arylation of pyridines, xanthenes, thiazole, pyrazine, and pyridazine^77^. Ru^2+^ was used as a photocatalyst in presence of water as solvent (aqueous formic acid in some cases) at room temperature. The photo source used was a 45 W fluorescent bulb. The Use of TEMPO suppressed the yield significantly, inferring that reaction proceeds via a radical pathway. Later on, in the same year, Lei and co-workers developed a new route for the total syntheses of menisporphine and daurioxoisoporphine using photoredox C–H arylation of isoquinoline as a key step (Fig. 5A, v)^78^. The reaction proceeded at room temperature under irradiation using a 40 W light source for 48 h. Malacria group reported the synthesis of biaryls using Ru(bpy)_3_Cl_2_.6H_2_O as photocatalyst^79^ under visible-light irradiation. Electron-withdrawing substituents on diazonium salts were found to be more efficient in terms of product formation. However, regioselectivity was found to be moderate. A novel protocol for the photocatalyzed C–H arylation of *N*-methylpyrrole, furan, thiophene, and their derivatives utilizing arylsulfonyl chlorides as coupling partners was reported by Bhasin and co-workers^80^. Arylsulfonyl chlorides are cheap, eco-friendly, and more stable compared to diazonium salts. The reaction took place in the presence of 3 mol% of [Ru(bpy)_3_]Cl_2_ under blue LED irradiation. The addition of TEMPO to the photocatalytic reaction inhibited the arylation process drastically, suggesting that the reaction went through a single electron transfer mechanism. Lee group developed the first aryl–aryl coupling by dual gold and photoredox catalysis, which does not require a stoichiometric amount of oxidants (Fig. 5A, vi)^81^. Ru(bpy)_3_(PF_6_)_2_ served as a photocatalyst with acetonitrile as solvent. Mesitylene derivatives were predominantly arylated using aryldiazonium salt at room temperature under blue LED irradiation. Regioselectivity was driven via the gold-catalyzed C–H activation step. Au(I)/Au(III) species were involved in catalysis, and the reaction followed a single electron transfer path. Photoredox catalyzed mono-selective *ortho*-C–H arylation of 6-arylpurine nucleosides was developed by Guo and co-workers in 2017^82^. The reaction conditions demonstrated strong functional group tolerance to a variety of aryldiazonium salts and 6-arylpurines derivatives. Photocatalyst was able to generate aryl radical and enabled a Pd(II)-Pd(III)-Pd(IV)-Pd(II) catalytic cycle, which in turn excluded the use of any external oxidants. Balaraman group illustrated another protocol for direct arylation of anilides^83^. This method was quite eco-friendly since it utilized a carbon dioxide-derived green solvent dimethyl carbonate (DMC) (Fig. 5A, vii). They performed mono- and di-arylation under visible-light dual catalysis and synthesized boscalid, a fungicide. Again, a well-known Ru/Pd dual catalytic system was used for visible-light C–H arylation of phenylurea using aryldiazonium salts^84^. Blue LED was used in this protocol for photon irradiation. Trapping experiments showed that the reaction went through a radical mechanism. Interestingly, this protocol was free from any external oxidants and additives.

In 2021, the Ackerman^85^ and the Greaney^86^ group independently reported *ortho*-C–H arylation of 2-arylazine derivatives at room temperature using visible-light ruthenium catalysis. In Ackerman protocol, [Ru(OAc)_2_(*p*-cymene)] in 1,4-dioxane was found to be the most effective combination (Fig. 5A, viii). Carboxylate-assisted C–H ruthenation followed by dissociation of *p*-cymene generated photocatalytically active biscyclometalated ruthenacycle from which an inner-sphere electron transfer took place. Methodology was extended to synthetically useful pyrazoles, triazoles, and sensitive nucleosides and nucleotides.

### Organophotocatalyzed C(sp^2^)–H arylation

**Fig. 6:**
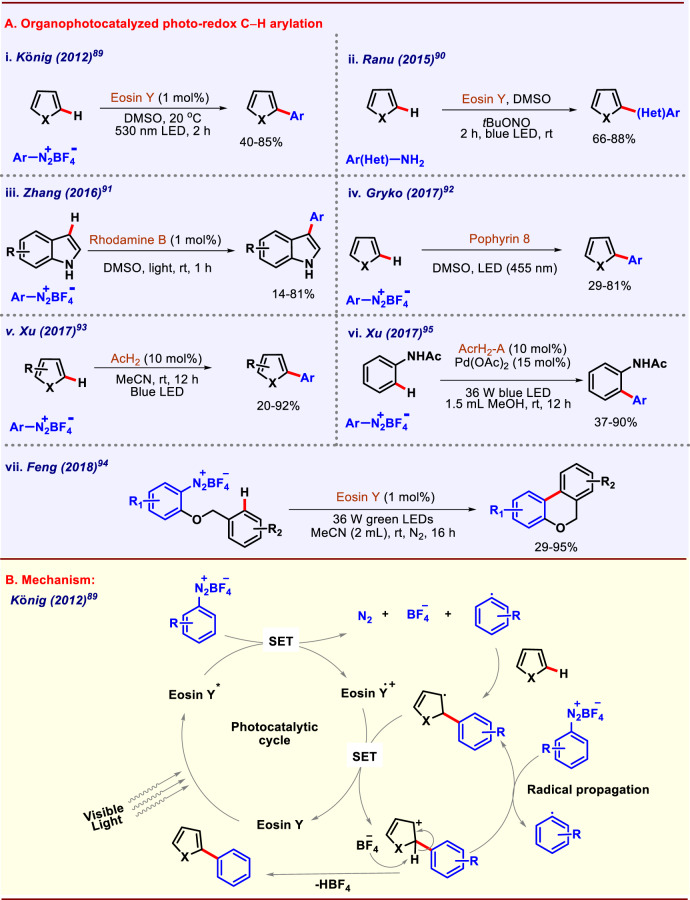
Photoredox catalyzed C–H arylation. **A** Organophotocatalyzed C–H arylation. **B** Mechanism of Eosin Y mediated C–H arylation of heteroarenes.

The efficiency of organic dyes as a photosensitizer to replace expensive Ru and Ir photocatalysts has been adopted by the synthetic community as well as industry for years. Organophotocatalyst provides cost-effective option for SET reactions, however, due to the short lifetime of excited state, high catalytic loading, and prolonged irradiation times are generally required. Cyanoarenes, benzophenones, quinones, acridinium, pyrylium, xanthenes, thiaznes, etc. are some common structural chromophores present in organic photoredox catalysts^87,88^. However, the most predominantly used organophotocatalyst is Eosin Y (Xanthenes). König group was the first to report C–H arylation of heterocycles using eosin Y as photocatalyst in metal-free conditions (Fig. 6A, i)^89^. The reaction proceeded by irradiation from a 530 nm green light source. The authors depicted the plausible mechanism for this single electron transfer transformation as shown (Fig. 6B). Ranu, Kundu, and co-workers developed a (hetero)arylation protocol by visible-light photocatalysis through the in situ generation of diazonium cation from (hetero)arylamines using *t-*BuONO at room temperature (Fig. 6A, ii). This was a novel green approach for C–H (hetero)arylation devoid of metal nitrites, high temperature, and acidic medium^90^. Surprisingly, 2-ethynylanilines derivatives underwent heteroarylation, which was not achieved by previously known methods. Different other photocatalysts like Rhodamine B, porphyrin, etc. were also used for C**–**H arylation reactions in metal-free conditions. Metal-free C-3 arylation of indole derivatives was performed by Zhang, Yang, and co-workers in photocatalytic conditions^91^. This approach lacked high temperatures, ligands, and transition-metal catalysts. The reaction was catalyzed by Rhodamine B under visible-light irradiation at room temperature (Fig. 6A, iii). Porphyrin-catalyzed photochemical arylation of heteroarenes was demonstrated by Gryko and co-workers in 2017 (Fig. 6A, iv)^92^. In this protocol, porphyrins get excited under light irradiation to perform redox activities. However, the redox property of the porphyrin system can be tuned by changing the substituents present at the periphery of the macrocycle. Optimization studies suggested that porphyrin H_2_T(F_5_P)P was the best among others. Xu group developed direct arylation of (hetero)arenes catalyzed by an organo-photoredox catalyst AcrH_2_ (NADH coenzyme model compound) using aryldiazonium salt as arylating reagents under visible-light irradiation. (Fig. 6A, v)^93^. Apart from thiophenes, furan, and pyrrole, they also reported C–H photoarylation of benzene. KIE value of 1.13 suggested that C–H cleavage of the benzene might not be the rate-determining step. The reaction was expected to follow a radical pathway which was confirmed by isolation of TEMPO-trapped intermediate. A Pschoor-type reaction was developed by Feng group in 2018 where they synthesized 6*H*-benzo[*c*]chromes through intramolecular arylation of arenediazonium salts via visible-light photoredox catalysis (Fig. 6A, vii)^94^. The reaction took place in metal-free conditions using eosin Y as photocatalyst and 36 W green LEDs as the visible-light source under an inert atmosphere. Furans, thiophenes, pyrroles underwent arylation at C-2 position without any requirement of metal catalyst. It is observed that sometimes only organic photocatalysts are not capable of selectively functionalizing a C–H bond. In those cases, a merging of metal catalyst is required. In 2017, Xu group performed *ortho*-C–H arylation of acetanilides^95^ by merging palladium catalysis with AcrH_2_ as an organic photocatalyst under visible-light conditions (Fig. 6A, vi). Balaraman and co-workers also reported a dual catalytic approach driven C–H arylation of anilides under oxidant-free conditions^96^. Aryldiazonium tetrafluoroborates which were used as aryl coupling partners also worked as an internal oxidant via C–N_2_ bond cleavage.

### Photoredox C(*sp*^3^)–H arylation

**Fig. 7:**
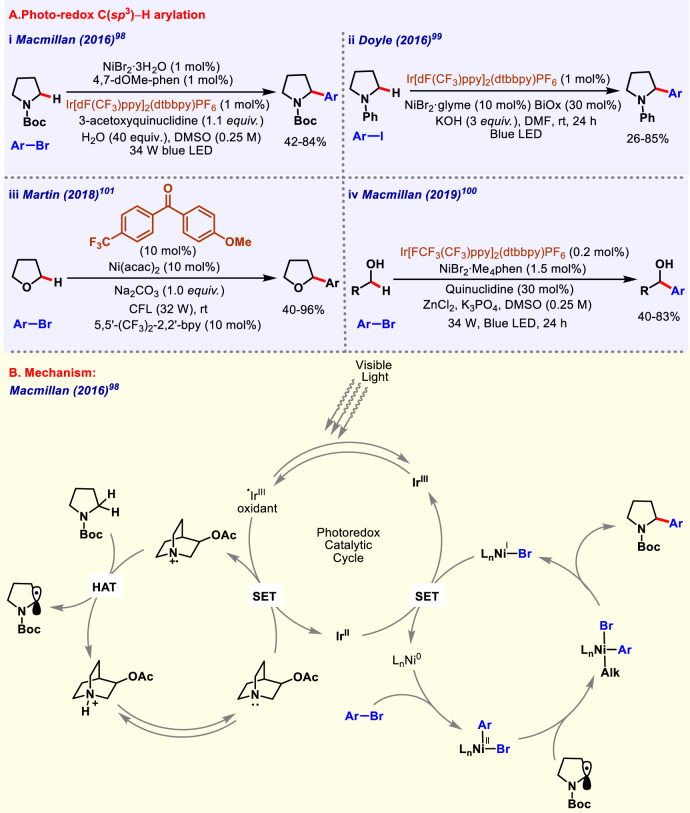
Photoredox catalyzed C(*sp*^3^)–H arylation. **A** Photoredox C(*sp*^3^)–H arylation of arenes and heteroarenes. **B** Reaction mechanism of Ni/Ir dual catalyzed photoredox catalysis.

Iridium and Nickel are well-established metal combination for forging carbon–carbon bonds via photoredox-mediated hydrogen atom transfer. HAT always been effective to generate radical intermediates. HAT catalysis capitalizes on inherit *sp*^3^-C–H bonds present in molecules for a variety of C–C bond constructions via reactions like Minisci reaction, conjugate addition, and radical–radical coupling. Notable contributions in this field are made by Macmillan, Martin, Doyle, Rovis groups, etc. Here we are discussing only some representative works performed in the field of C(*sp*^3^)–H arylation harnessing light energy. Direct C(*sp*^3^)–H arylation of amines carries pivotal importance, due to prevalence of saturated amines in organic synthesis. Amines are known to undergo C–H functionalization at α-position via formation of iminium cation or anion. Macmillan group in 2011 reported, α-arylation of *N*-phenyl amines with cyanoarenes as the coupling partner using iridium photocatalysis in white LED which proceeded through formation of iminium radical^97^. In 2016, the same group demonstrated Ir photoredox-mediated hydrogen atom transfer and nickel catalyzed selective arylation of α-amino and α-oxy C(*sp*^3^)–H bonds in both cyclic and acyclic systems (Fig. 7A, i)^98^. Various cyclic amines such as pyrrolidine, azetidine, piperidine, and azepane underwent selective C–H arylation. Heteroaryl chlorides were also found to be effective electrophiles in this protocol. Plausible catalytic cycle proposed by the authors is shown (Fig. 7B). In 2016, Doyle and co-workers also performed arylation of *N*-phenyl amines with the help of nickel–iridium photoredox catalysis and aryl iodides as the arylating agent (Fig. 7A, ii)^99^. Various reaction parameters were well optimized to get best possible results in terms of yield and selectivity. As a whole, they developed a mild protocol for synthesis of benzyl amines without any need of prefunctionalization. α-C–H arylation of free alcohols through iridium–nickel photoredox catalysis via HAT were developed by MacMillan (Fig. 7A, iii)^100^. Apart from Ir and Ni, zinc also played a crucial role; deprotonation of alcohol to activate α-C–H bond simultaneously suppressing C–O bond formation as well as also deactivated the other hydridic bonds present in the molecule. A 3-step synthesis of the drug Prozac was performed through this method. In 2018, Martin and co-workers devised a methodology for C(*sp*^3^)–H arylation of alkanes using aryl halides as starting precursors (Fig. 7A, iv)^101^. This reaction utilizes a combination of nickel catalysts and triplet excited ketones as photosensitizers, thus avoiding the use of widely used Iridium-based photosensitizer. Triplet diaryl ketones as catalyst were also responsible for C(*sp*^3^)–H bond activation. This protocol tolerated wide range of electron-deficient nitrogen-containing heterocycles as well as complex arenes containing chiral groups *viz* cholesterol, L-menthol, D-allofuranose, and D-phenylglycine.

### Electrochemical C–H arylation

Electrochemical C–H arylation came up as an advanced and efficient tool to enable C–C bond formation reactions in a greener and sustainable fashion^102^. Nowadays, electrochemistry is considered to be a promising atom economic alternative to traditional stoichiometric oxidants and reductants, hence preventing additional side reactions. Manual control of oxidative potential instead of relying on chemical oxidant is an additional feature of electrochemical transformations, which further enhances the scope of electrochemistry in C–H functionalizations. Thus, a joint venture of electrochemistry and transition-metal-catalyzed C–H functionalization is expected to improve chemo- and regioselectivity of C–H functionalization and is a rapidly growing area of interest that provides synthetic opportunities that could not be achieved by means of conventional chemistry.

### Electrochemical C(*sp*^2^)–H arylation of arenes and heteroarenes

**Fig. 8:**
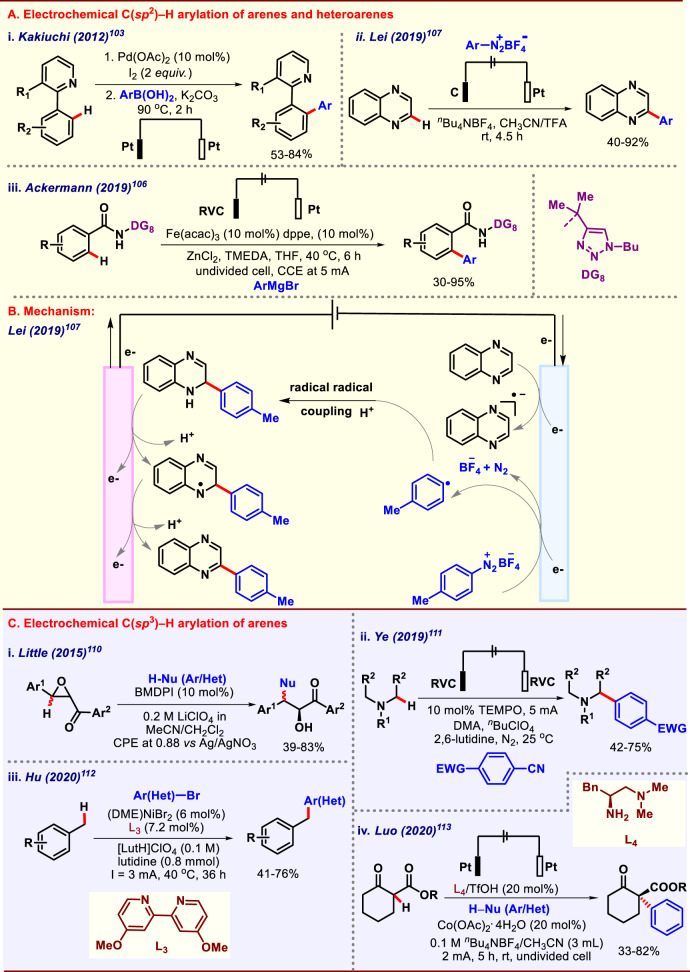
Electrochemical C–H arylation. **A** Electrochemical C(*sp*^2^)–H arylation of arenes. **B** Mechanism of electrochemical C–H arylation of arenes. **C** Electrochemical C(*sp*^3^)–H arylation of arenes.

In 2012, Kakiuchi and co-workers executed electerochemical C–H arylation that consisted of two steps. The first step being electrochemical iodination followed by Suzuki cross-coupling (Fig. 8A, i)^103^. Pd catalyzed electrochemical C–H iodination occurred exclusively at *ortho-*position, followed by Suzuki coupling to give the desired product. In 2016, Wan and co-workers developed a protocol for direct arylation of pyrroles via indirect electroreductive C–H functionalization^104^. Perylene-3,4,9,10-tetracarboxylic acid diimide (PDI) was employed as a redox mediator for this indirect electroreductive transformation. In the absence of PDI no desired product was obtained that reveals the importance of PDI in this electroreductive indirect transformation. Later in 2017, Charushin and co-workers also disclosed an atom and step economical nucleophilic arylation and heteroarylation of aza-aromatics via electrochemical oxidative C–C cross-coupling reaction^105^. This work was a fine example of C–H functionalization without metal catalyst that made this protocol economical and eco-friendly. In 2019, Ackermann and co-workers executed electrolysis enabled Fe catalyzed C–H arylation of benzamides triazoles (Fig. 8A, iii)^106^. Herein they reported DCIB (dichloroisobutane) free Fe catalyzed C–H arylation of benzamides triazoles, enabled by electricity as the sole oxidant, thus expanded the scope of Fe catalysts in terms of sustainability of transformation. Quinoxalines are an abundant structural motif present in many natural products and are an example of the electron-deficient arene. Earlier also, many efforts have been paid to C–H functionalization of electron-deficient arenes but transition-metal-catalyzed C–H activation of electron-deficient arenes produced environmentally deleterious waste and was also not economical at all. Therefore, an eco-friendly and economic methodology for C–H activation of electron-deficient arenes was required. In this regard, Lei and co-workers in 2019 reported an electrochemical arylation of electron-deficient arenes through reductive activation (Fig. 8A, ii)^107^. The reaction proceeds through the given plausible mechanism (Fig. 8B). Lei and co-workers again in the same year developed another protocol for electrochemical deoxygenative C-2 arylation of quinolines-*N*-Oxides employing sulfonylhydrazines as the other coupling partner^108^. Electricity playing the dual role of oxidant and reductant that magnifies the scope of this methodology in terms of sustainability. Very recently, Ackermann and co-workers reported unprecedented manganese-electrocatalyzed C–H arylations assisted by weakly co-ordinated directing group^109^. This protocol did not require the presence of zinc additive as it was required in their previous iron-electrocatalyzed work to prevent formation of homocoupled product of Grignard reagent. Various transition metals including 4d metals like palladium were tested for this job. However, no product formation or trace amount of product was observed with iron catalyst. With optimal condition in hand, various aryl motifs were successfully incorporated into benzamides in good yields.

### Electrochemical C(*sp*^*3*^)–H arylation

Little and co-workers in 2015 performed electrochemically enabled Friedel Crafts type of arylation of chalcone epoxides catalyzed by triarylimidazole redox mediator (Fig. 8C, i)^110^. 2-(4-Bromophenyl)-1-methyl-4,5-diphenyl-1H-imidazole (BMDPI) earlier also was employed for benzylic C–H functionalization; herein they diversified the scope of BMDPI’s by successfully employing them in the arylation of chalcone epoxides. An ample substrate scope was performed, and various heteroarenes were incorporated into chalcone epoxides with only a stoichiometric amount of electricity. Benzyl amines are ubiquitous structural motifs; owing to their high abundance in natural products and medicinal agents, an efficient synthesis of benzylamine was highly desired. In this regard, Ye and co-workers in 2019 reported direct arylation of *α*-amino C(*sp*^3^)–H bonds by convergent paired electrolysis (Fig. 8C, ii)^111^. Later in 2020, Hu and co-workers reported direct arylation of benzylic C–H bonds enabled by Ni catalyst and convergent pair electrolysis (Fig. 8C, iii)^112^. Ni catalyst is acting like a thread that binds with both the intermediates and enables cross-coupling between them to give the desired product. The presence of fluorine-doped tin oxide-coated glass as an anode was also essential for the successful completion of this transformation. The scope of electrolysis in asymmetric synthesis has mostly remained underexplored due to the preconceptions that ionic media can disfavour the mechanism and scope of asymmetric synthesis. Another reason for the underdevelopment of asymmetric electrochemical reaction can be attributed to the tremendous success of thermal and photochemical analogs that overshadowed the electrochemical version. However, the increased demand for greener and economical methodologies compelled chemists to explore in the direction of asymmetric electrochemical transformations. In this regard, Luo and co-workers reported the very first asymmetric *α*-arylation of cyclic *β*-ketoester with the help of chiral amine catalyst through an electrochemical pathway (Fig. 8C, iv)^113^. 1-Aminobenzotriazole was used as the benzyne precursor to couple with ethyl 2-oxocyclohexanecarboxylate in the presence of chiral amine catalyst. Another issue to address was selective capture of in situ generated benzyne by the catalytic amine intermediate in preference over other reactive species present in media. To resolve this problem Co(OAc)_2_.4H_2_O was used as an additive in a simple electrochemical system consisting of Pt-electrodes in an undivided cell. Gratifyingly, by this catalytic system, moderate yield and good enantiomeric excess of the desired product were obtained.

### C–H arylation in flow chemistry

**Fig. 9:**
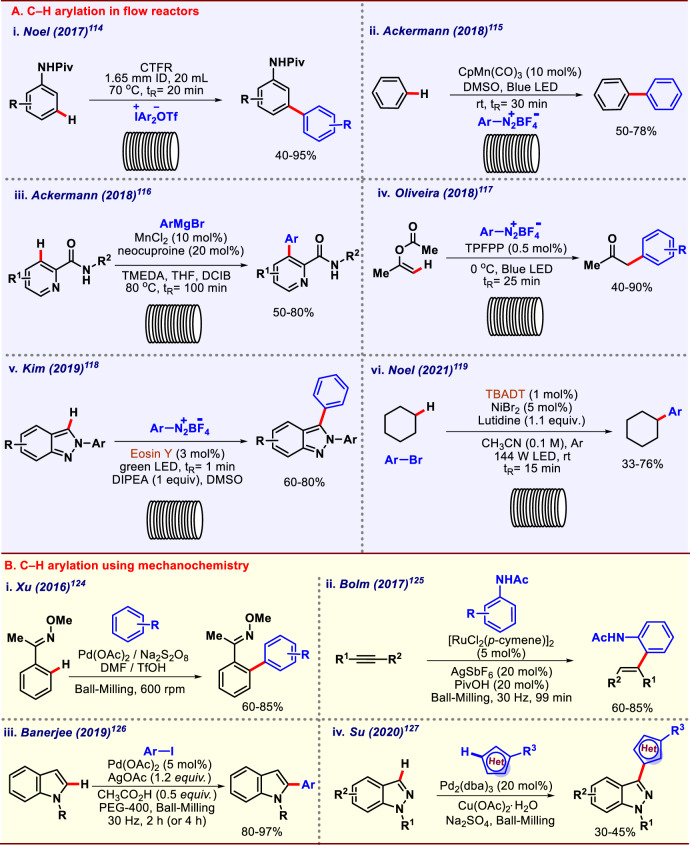
Arylation utilizing continuous flow processes and mechanochemistry. **A** C–H arylation in continuous flow process. **B** Mechanochemical C–H arylation.

Continuous flow processes are flourishing among chemists as it solves many commonly encountered problems faced during synthesis such as handling of hazardous chemicals, implicit or explicit sluggish nature of reactions which encouraged chemists to carry out transition-metal-catalyzed C–H transformations in flow setup to increase the applicability of C–H activation. Keeping the advantages in mind, Noel and co-workers in 2017 reported C–H arylation of anilines in flow reactors, a much faster, safer, and superior protocol over the earlier batch one (Fig. 9A, i)^114^. This design of the setup included the use of four continuous flow modules that can be operated either separately or individually to provide direct access to *meta*-arylated anilines. A broad substrate scope was studied, diverse functional groups with electronically different properties were well-tolerated that reflects the synthetic potential of this protocol. Photochemistry was merged with flow to achieve cleaner product formation without harsh reaction conditions that too in a reduced time scale. In 2018, Ackermann and co-workers reported manganese catalyzed C–H arylation in flow setup merging with blue LED (Fig. 9A, ii)^115^. Superior yields as compared with batch processes and use of earth-abundant transition metal as catalyst were some major merits of the established protocol. An ample substrate scope was obtained, showing the robustness of the methodology. Later in the year, the same group reported another fine example of C–H arylation in flow by employing MnCl_2_ as catalyst (Fig. 9A, iii)^116^. The unique performance level of manganese catalyst was revealed by the incompatibility of other metals catalysts like iron, copper, ruthenium, and palladium. Oliveira and co-workers reported photoredox C–H arylation in flow, enabled by porphyrins as photocatalyst. The C(*sp*^*2*^)–H arylation of enol-acetates produced *α*-arylketone or *α*-arylaldehyde as the desired product (Fig. 9A, iv)^117^. A significant difference in yields was also observed in batch versus flow that clearly displayed the superiority of flow processes. Later in 2019, Kim and co-workers reported visible-light-mediated direct arylation of indazoles, enabled by eosin Y as the photocatalyst (Fig. 9A, v)^118^, which was quite economical and attractive approach. Very recently in 2021, Noel and co-workers reported C(*sp*^3^)–H arylation in flow enabled by nickel and TBADT (tetrabutylammonium decatungstate) catalysis (Fig. 9A, vi)^119^.

Herein they clearly manifested the potential of flow chemistry over conventional batch catalysis. In the flow setup, not only reactions got accelerated, but also better yields were observed as compared to batch reactions.

### C–H arylation in mechanochemistry

In the past few decades, chemists have really strived for development of new and cleaner synthetic methodologies. Owing to this surge of new and cleaner transformations, a resurgence has been witnessed in solid-state chemical reactions that provided crucial benefits over conventional ones in dissolved state. The chemical transformations promoted by exerting mechanical stress and shear fall in the genre of mechanochemistry. Mechanochemistry term was introduced by Wilhelm Ostwald and he described it as a branch of chemistry which is concerned with chemical and physio-chemical changes of substances of all states of aggregation due to the influence of mechanical energy. Mechanochemistry avoids the use of solvents and also works on lower energy demands, therefore is cheaper as compared with other traditional approaches. Various conventional organic reactions like Aldol condensation, Wittig olefination, Michael addition along with some transition-metal-catalyzed transformations are well-established in mechanochemistry^120–123^. The C–H activation embedded with mechanochemistry offered some great advantages *viz*. milder reaction conditions, residue-free product formation, shorter reaction length, and use of the lesser quantity of solvent or solvent-free approach. These particular features of mechanochemistry encouraged chemists to explore the applicability of C–H transformations in mechanochemistry. In 2016, Xu and co-workers reported mechanochemical dehydrogenative C–H arylation using oxime moiety as directing group (Fig. 9B, i)^124^. Earlier, similar work was performed by few other groups in batch fashion. However, it came with several shortcomings like harsh reaction conditions, low functional group tolerance, and longer reaction time. Xu and co-workers envisioned a mechanochemical protocol to remove all these shortcomings of batch reaction. Various functional groups were well-tolerated on both the coupling partners, and almost exclusive regioselectivity was observed for *para*-C–H functionalization of arene that indicated the usefulness of this mechanochemical transformation. In 2017, Bolm and co-workers also presented a fine example of hydroarylation of alkyne using mechanochemistry (Fig. 9B, ii)^125^. The formed alkenylated acetanilide was transformed into indoles by means of a palladium-catalyzed annulation reaction to magnify the synthetic scope of this protocol. The application of the catalytic amount of oxidant, as well as solvent was noteworthy. Indoles are an important heterocyclic compound present in many bioactive compounds and also in pharmacophores; therefore, arylation of indoles has paramount importance in an aspect of API (Active Pharmaceutical Ingredient) syntheses. Already a number of methodologies for arylation of indoles are well-known, either by cross-coupling or by C–H activation but all of them have several shortcomings. Some had limited substrate scope or some required super stoichiometric amount of silver oxidant. Therefore, a perfect method for arylation of indoles was still elusive. In this context, Banerjee and co-workers in 2019 reported mechanochemical arylation of indoles (Fig. 9B, iii)^126^. Diverse functional groups with electronically varying properties were well-tolerated, showing the robustness of this mechanochemical protocol. Later, Su and co-workers in 2020 reported mechanochemical C–H arylation of indazoles (Fig. 9B, iv)^127^. Indazoles being an abundant structural motif present in many pharmaceuticals. Heteroarylation of indazoles carried a lot of potential. Furan and thiophene derivatives were well-tolerated under this protocol as well.

## Conclusion and future outlook

Transition-metal-catalyzed C–H arylation has emerged as a desirable alternative to traditional C–C coupling reactions. DG-assisted functionalization is quite important from industrial point of view due to the high selectivity of the desired products. Recently, chemists came up with new design of ligands to promote non-directed C–H arylation that does not require any directing auxiliary. However, this approach is limited to only certain type of substrates and therefore need further ligand engineering. C–H arylation techniques need further refinement to make the process more robust toward natural product synthesis, drug discovery, and agrochemicals production. There is still plenty of room in the advancement of photoredox C–H arylation at distal *sp*^*2*^ & *sp*^*3*^ C–H bonds. Recent progress in the artificial metalloenzymes catalyzed enantioselective C–H functionalization unlocks a new path toward the exploration of asymmetric C–H arylation. Artificial metalloenzyme-driven C–H arylation will help to imitate the natural metalloenzyme which in turn can be utilized for targeted drug delivery. Enantioselective C–H arylation via the merger of organo-, electro-, and photoredox catalysis is still elusive. Development of effective 3d metal-based catalysts is necessary for the growth of electrochemical C–H arylation. Modern mechanochemical techniques need further modifications with respect to energy consumption, temperature, and other parameters to make it viable for industrial-scale applications. Such environment benign protocols will help the industries to inculcate solvent-free approaches toward the synthesis of biaryl drug precursors. Handling of heterogeneous reaction mixture is always challenging in continuous flow processes, so chemists around the globe need to come up with modifications in traditional reaction processes to make more batch processes viable in flow reactors. To address these inadequacies, development of new catalytic system, ligands design, and merger of greener methodologies are vital for sustainable development.
